# Thickness ranges calculation method of double asphalt overlay on concrete pavement

**DOI:** 10.1038/s41598-024-54827-x

**Published:** 2024-02-28

**Authors:** Xiang Fu, Jiayu Huang, Yuxin Ban, Jun Duan, Jianuo Xie

**Affiliations:** 1 School of River and Ocean Engineering, Chongqing Jiaotong University, Chongqing, 400074 China; 2 School of Civil Engineering and Architecture, Chongqing University of Science and Technology, Chongqing, 401331 China; 3 School of Civil Engineering, Chongqing University, Chongqing, 400044 China; 4 College of Traffic and Transportation, Chongqing Jiaotong University, Chongqing, 400074 China

**Keywords:** Asphalt overlay, Thickness, Shear stress, Mohr–Coulomb criterion, Tack coat, Civil engineering, Techniques and instrumentation

## Abstract

Asphalt overlay is widely used in maintaining and rehabilitating highway system performance. However, explicit calculation methods for the asphalt overlay thickness range is lacking. Taking stone mastic asphalt (SMA) and asphalt concrete (AC) asphalt overlay on cement concrete pavement as examples, the paper proposed a design method for the asphalt overlay thickness range based on the shear performance of the interlayer. Firstly, the shear stress distribution regularities on the asphalt overlay and Portland cement concrete interlayer was calculated with a multilayer elastic theory. Meanwhile, the shear strength was obtained from a series of direct shear tests. The shear characters of the asphalt overlay met with the Mohr–Coulomb criterion, and the shear strength parameters cohesive force $c$ and interface friction angle $\varphi$ on the interlayer were acquired. Finally, a method for determining the thickness range of double layer asphalt overlay under different traffic conditions was given. The epoxy resin adhesive was recommended for the highway with severe local premature shear failure compared with the modified emulsion asphalt. Therefore, through the above research, the amount of asphalt used is controlled in a reasonable range, thus improving the pavement structure durability and reducing energy consumption.

## Introduction

Asphalt mixture surfacing is used worldwide to replace original Portland cement concrete (PCC) worn surface as a sustainable rehabilitation strategy to improve pavement performance and reduce the cost of maintenance^1,2^. In the past few decades, asphalt overlay experienced development from traditional dense-graded mixture overlays to thin gap-graded mixtures overlays (such as stone mastic asphalt, SMA), and to a very thin asphalt overlay. According to the World Road Association^3^, the traditional dense-graded mixture overlays is about 50 mm, the thin gap-graded mixtures overlay is 30–50 mm, and the very thin asphalt overlay is 20–25 mm. Until now, thin asphalt overlay pavement is widely in service for the Chinese highway network. However, premature failure is usually observed with distresses such as slippage cracking, delamination and potholes.

Damage occurred on the asphalt mixture surfacing problems are studied to be associated with shear properties of pavement structure induced by insufficient thickness^4^. Related research on the shear performance of pavement structure mainly focuses on the asphalt overlay and asphalt–asphalt interface, studies on asphalt–concrete interface are limited. There are significant differences in the mechanical performance between asphalt–asphalt interface and asphalt–asphalt interface, as former is flexible–flexible contact and the latter is flexible–rigid contact. Ge et al.^5^ proposed that shear property of interlayer between the asphalt overlay and old PCC pavement slab reaches optimum with glass fiber reinforced modified asphalt membranes, but it will increase the project cost. Interfacial shear properties depend on various factors, such as mixture material, temperature, the thickness of each material layer, vehicle load, tire type, bonding conditions^6^. Hu and Walubita^7^ analyzed the mechanistic responses of tensile, compressive, and shear stresses under measured vertical and assumed horizontal tire-pavement contact pressure. Hu et al.^8^ studied the effect of tack coat dosage and type, as well as four temperature gradients on interfacial shear properties, and they concluded that the interfacial shear properties decreased sharply as the temperature increased. Wan et al.^9^ further tested the shear characteristics of asphalt pavement interface, and they believed that the pavement served for 4 years had a risk of distress due to the interface shear characteristics loss.

An extensive investigation was launched as early as the mid-1970s, and researchers agreed that increasing asphalt pavement thickness could reduce structural distress, such as fatigue crack and rutting^10–12^. It is critical to recognize that there should be a threshold for the thickness value. According to Nunn and Ferne^13^ the asphalt layer thickness of 15.4 in. was enough, and no more thickness was necessary from the aspect of long pavement life. Wang^14^ developed a predictive model of pavement thickness based on logistic regression equation of asphalt concrete overlay cracking. Bianchini et al.^15^ proposed an improved methodology for calculating the minimum overlay thickness by introducing a correction factor of quality and structural condition. Wang^16^ reported an optimal thickness calculation method of pavement asphalt layer based on energy dissipation considering the environment and other parameters. Most of the current asphalt surface is designed from the aspect of controlling the reflective cracking by considering the deflection values and flexural stress^17^. However, the problem of interlayer shear strength is not taken into account, and the actual problems of pavement layer pushing, shedding, and wrapping diseases are indeed induced by interlayer shear.

The paper aims at proposing a new calculation methodology for the reasonable overlay thickness range of the asphalt mixture surfacing on concrete pavement under different traveling conditions by discussing the interfacial shear resistance capacity between the asphalt overlay and the old PCC slab. The study is performed on a common used pavement structure in Chongqing, China, i.e., the SMA-13 (stone mastic asphalt-13) asphalt overlay and AC-16I (asphalt concrete-16I) overlaying PCC pavements. First, the distribution pattern of shear stress under different influencing factors (asphalt overlay thickness, friction coefficient on the wearing course, vehicle load and interfacial bonding condition) is studied with the multilayer elastic theory. Furthermore, direct shear tests are designed to investigate interfacial shear properties on the laboratory scale. Finally, shear strength envelope curves are compared with maximum shear stress curves to establish the reasonable calculation method of asphalt overlay thickness range.

## Mechanics theory

### Multilayer elastic theory

**Figure 1:**
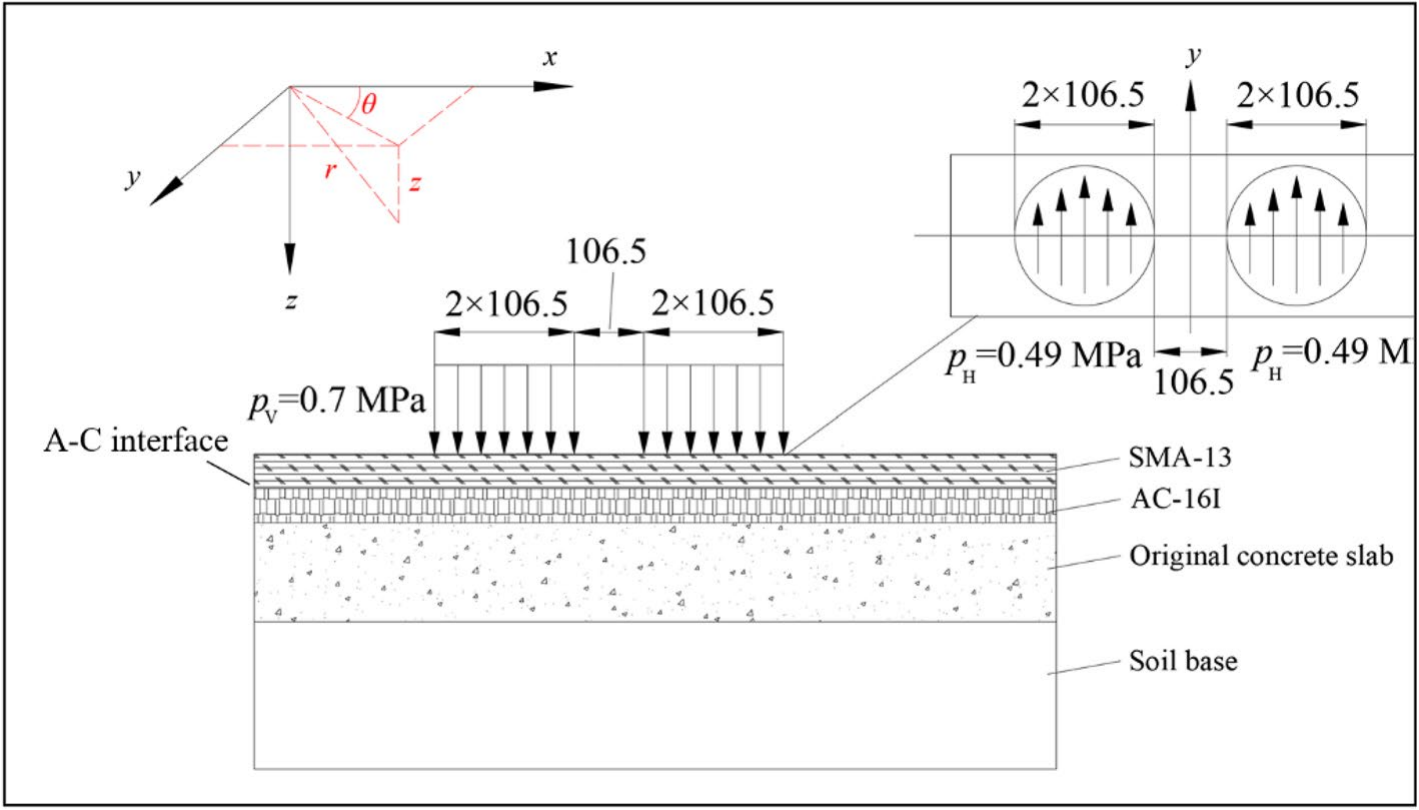
Typical double-layered pavement system model and vehicle wheel load mechanical analysis. (Under standard vehicle load $p_{V} = 0.7 \, MPa$) (unit: mm, A–C interface is short for AC–16I and PCC interface).

Figure 1 shows a typical double-layered highway model composed of SMA-13, AC-16I, original PCC slab, and soil base successively. The multilayer elastic theory^18^ is introduced in the cylindrical coordinate system ($r,\theta ,z$). The normal stress $\sigma_{z}$ and shear stress $\tau_{rz}$ of the asphalt pavement system can be calculated. The normal stress $\sigma_{z}$ is used for later shear strength calculation with Mohr–Coulomb criterion, and the shear stress $\tau_{rz}$ is used for calculating the shear stress. The method is developed based on the following hypotheses: (a) The pavement consists of a homogeneous isotropic linear elastic material; (b) The soil base is semi-infinite in both horizontal (*y*) and vertical (*z*) direction; each pavement layer is limited in the *z*-direction and infinite in semi-longitudinal (*x*) and (*y*) direction; (c) Wheel load is uniformly distributed within a circular area; (d) Self-weight of each layer is not considered.

The vehicle wheel pressure acting on asphalt pavement can be divided into vertical axisymmetric load and longitudinal load.

1. Vertical axisymmetric load.

Here, the *z-*axial is set as a symmetry axis, and the superscript *V* represents the vertical axisymmetric load acting. For spatial axisymmetric load, shear stress in two directions $\tau_{\theta z}^{V} = \tau_{r\theta }^{V} = 0$, the deformation compatibility equation is:

1 $$\left\{ \begin{gathered} \nabla^{2} \sigma_{r}^{V} - \frac{2}{{r^{2} }}(\sigma_{r}^{V} - \sigma_{\theta }^{V} ) + \frac{1}{1 + \mu }\frac{{\partial^{2} \Theta }}{{1 + \mu \partial^{2} r^{2} }} = 0 \hfill \\ \nabla^{2} \sigma_{\theta }^{V} - \frac{2}{{r^{2} }}(\sigma_{r}^{V} - \sigma_{\theta }^{V} ) + \frac{1}{1 + \mu }\frac{\partial \Theta }{{r\partial r}} = 0 \hfill \\ \nabla^{2} \sigma_{r}^{V} + \frac{1}{1 + \mu }\frac{{\partial^{2} \Theta }}{{\partial^{2} z^{2} }} = 0, \hfill \\ \nabla^{2} \tau_{zr}^{V} - \frac{{\tau_{zr}^{V} }}{{r^{2} }} + \frac{1}{1 + \mu }\frac{{\partial^{2} \Theta }}{\partial r\partial z} = 0 \hfill \\ \end{gathered} \right\},$$

where, $\nabla^{2} = \frac{{\partial^{2} }}{{\partial r^{2} }} + \frac{1}{r}\frac{\partial }{\partial r} + \frac{1}{{r^{2} }}\frac{{\partial^{2} }}{{\partial \theta^{2} }} + \frac{{\partial^{2} }}{{\partial z^{2} }}$ is the Laplace operator, and $\Theta = \sigma_{r}^{V} + \sigma_{z}^{V} + \sigma_{\theta }^{V}$ is the first stress invariant.

By using the Love stress function $\varphi = \varphi \left( {r,z} \right)$, we can get stress components under vertical axisymmetric load:

2 $$\left\{ \begin{gathered} \sigma_{z}^{V} = \frac{\partial }{{\partial_{z} }}\left[ {\left( {2 - \mu } \right)\nabla^{2} \varphi - \frac{{\partial^{2} \varphi }}{{\partial z^{2} }}} \right] \hfill \\ \tau_{zr}^{V} = \frac{\partial }{{\partial_{r} }}\left[ {\left( {1 - \mu } \right)\nabla^{2} \varphi - \frac{{\partial^{2} \varphi }}{{\partial z^{2} }}} \right] \hfill \\ \end{gathered} \right\},$$

where, ${\sigma }_{z}^{V}$ and ${\tau }_{zr}^{V}$ are normal stress and shear stress under vertical axisymmetric load.

Taking Eq. (2) into Eqs. (1) and (3),

3 $$\left\{ \begin{gathered} \frac{{\partial \sigma_{x} }}{{\partial_{x} }} + \frac{{\partial \tau_{xy} }}{{\partial_{y} }} + \frac{{\partial \tau_{xz} }}{{\partial_{z} }} = 0 \hfill \\ \frac{{\partial \tau_{xy} }}{{\partial_{x} }} + \frac{{\partial \sigma_{y} }}{{\partial_{y} }} + \frac{{\partial \tau_{yz} }}{{\partial_{z} }} = 0 \hfill \\ \frac{{\partial \tau_{xz} }}{{\partial_{x} }} + \frac{{\partial \tau_{yz} }}{{\partial_{y} }} + \frac{{\partial \sigma_{z} }}{{\partial_{z} }} = 0 \hfill \\ \end{gathered} \right\}.$$

We get the biharmonic equation $\nabla^{2} \nabla^{2} \varphi = 0$, which can be solved by Hankel integral transformation:

4 $$\varphi (r,z) = \int_{0}^{\infty } {\left[ {(A_{\zeta } + B_{\zeta } z)e^{ - \zeta z} + \left( {C_{\zeta } + D_{\zeta } z} \right)e^{\zeta z} } \right]} \zeta J_{0} \left( {\zeta r} \right)d_{\zeta } ,$$

where, $J_{0} \left( {\zeta r} \right)$ is the zero-order Bessel function. $A_{\zeta } ,B_{\zeta } ,C_{\zeta }$ and $D_{\zeta }$ are parameters that have to be determined with the boundary conditions and continuous conditions between layers. Taking Eq. (4) into Eq. (2), stress and displacement components are got:

5 $$\left\{ \begin{gathered} \sigma_{z}^{V} = \int_{0}^{\infty } {\zeta \left\{ {\left[ {A + (1 - 2\mu + \zeta z)B} \right]e^{ - \zeta z} - \left[ {C - (1 - 2\mu - \zeta z)D} \right]e^{\zeta z} } \right\}J_{0} (\zeta r)} d_{\zeta } \hfill \\ \tau_{zr}^{V} = \int_{0}^{\infty } {\zeta \left\{ {\left[ {A - (2\mu - \zeta z)B} \right]e^{ - \zeta z} + \left[ {C + (2\mu + \zeta z)D} \right]e^{\zeta z} } \right\}J_{1} (\zeta r)d_{\zeta } } \hfill \\ u = - \frac{1 + \mu }{E}U \hfill \\ \end{gathered} \right\},$$

where, $U = \int_{0}^{\infty } {\left\{ {\left[ {A - \left( {1 - \zeta z} \right)B} \right]e^{\zeta z} - \left[ {C + \left( {1 + \zeta z} \right)D} \right]e^{\zeta z} } \right\}} J_{1} \left( {\zeta r} \right)d_{\zeta }$. For the *n*-layered elastic system, the boundary conditions are:

6 $$\left\{ \begin{gathered} \sigma_{z}^{V} \left| {_{z = 0} } \right. = \left\{ \begin{gathered} - p\left( r \right),{\text{when }}r \le \sigma \\ \mathop {}\nolimits 0\mathop {}\nolimits ,{\text{when }}r > \sigma , \\ \end{gathered} \right. \hfill \\ \tau_{zr}^{V} \left| {_{z = 0} = 0} \right. \hfill \\ \end{gathered} \right.$$

and the interlayer contact conditions are:

7 $$\begin{gathered} \left[ {\sigma_{z}^{V} } \right]_{i} = \left[ {\sigma_{z}^{V} } \right]_{i + 1} , \, \left[ {\tau_{zr}^{V} } \right]_{i} = \left[ {\tau_{zr}^{V} } \right]_{i + 1} {\text{ (Fully bonded)}} \hfill \\ \left[ {\sigma_{z}^{V} } \right]_{i} = \left[ {\sigma_{z}^{V} } \right]_{i + 1} , \, \left[ {\tau_{zr}^{V} } \right]_{i} = \left[ {\tau_{zr}^{V} } \right]_{i + 1} {\text{ = 0 (Complete unbonded)}} \hfill \\ \left[ {\sigma_{z}^{V} } \right]_{i} = \left[ {\sigma_{z}^{V} } \right]_{i + 1} , \, \left[ {\tau_{zr}^{V} } \right]_{i} = \left[ {\tau_{zr}^{V} } \right]_{i + 1} { = }K_{i} \left( {u_{i + 1} - u_{i + 1} } \right){\text{ (Semi - bonded)}} \hfill \\ \end{gathered}$$

where, $K\left( {N/cm^{{3}} } \right)$ is the interfacial bonding coefficient between the *i*th and (*i* + 1)^th^ layers, $0 \le K < \infty \, (i = 1,2,...,n - 1)$.

Finally, stress induced by the external load is zero for the *n*th layer, thus $C_{n} = D_{n} = 0$.

(2) Longitudinal load.

It's a non-axisymmetrical problem for the elastic system imposed by longitudinal vehicle load. The Michel stress function φ and ψ, satisfying the second-order partial differential equation, $\nabla^{2} \varphi = 0$ and $\nabla^{4} \psi = \nabla^{2} \nabla^{2} \psi = 0$ are used:

8 $$\left\{ \begin{gathered} \sigma_{z}^{L} = \frac{\partial }{\partial z}\left[ {\left( {2 - \mu } \right)\nabla^{2} \psi - \frac{{\partial^{2} \psi }}{{\partial z^{2} }}} \right] \hfill \\ \tau_{zr}^{L} = \frac{\partial }{\partial r}\left[ {\left( {1 - \mu } \right)\nabla^{2} \psi - 2\frac{{\partial^{2} \psi }}{{\partial z^{2} }}} \right] + \frac{1}{r}\frac{{\partial^{2} \varphi }}{\partial \theta \partial z} \hfill \\ \end{gathered} \right\},$$

where, the superscript *L* represents the longitudinal load acting, and the Laplace operator is $\nabla^{2} = \frac{{\partial^{2} }}{{\partial r^{2} }} + \frac{1}{r}\frac{\partial }{\partial r} + \frac{1}{{r^{2} }}\frac{{\partial^{2} }}{{\partial \theta^{2} }} + \frac{{\partial^{2} }}{{\partial z^{2} }}$.

Stress components can be obtained with Hankel integral transformation:

9 $$\left\{\begin{array}{c}{\sigma }_{\text{z}}^{\text{L}}\text{=}\left[{\int }_{0}^{\infty }{\zeta }^{3}{{\text{J}}}_{1}\text{(}\zeta {\text{r}}\text{)}\left\{\left[\zeta {\text{A}}_{\text{i}}\text{-(1-2}\mu \text{+}\zeta {\text{z}}\text{)}{\text{B}}_{\text{i}}\right]{\text{e}}^{-\zeta {\text{z}}}-\left[\zeta {\text{C}}_{\text{i}}\text{-(1-2}{\mu }_{\text{i}}-\zeta {\text{z}}\text{)}{\text{D}}_{\text{i}}\right]{\text{e}}^{\zeta {\text{z}}}\right\}{\text{d}}_{\zeta }\right]cos\theta \\ {\tau }_{zr}^{L}=\left[{\int }_{0}^{\infty }{\zeta }^{3}{J}_{1}(\zeta r)\left({E}_{i}{e}^{-\zeta z}+{F}_{i}{e}^{\zeta z}\right){d}_{\zeta }-\frac{1}{r}{U}_{2}\right]sin\theta \end{array}\right\},$$

where, ${\sigma }_{z}^{L}$ and ${\tau }_{zr}^{L}$ are normal stress and shear stress under longitudinal load. $J_{1} (\zeta r)$ is the first-order Bessel function, and

10 $$U_{2} = - \int_{0}^{\infty } {\zeta^{2} } J_{2} \left( {\zeta r} \right)\left\{ {\left[ {\zeta A_{i} - \left( {1 - \zeta z} \right)B_{i} - 2E_{i} } \right]e^{ - \zeta z} - \left[ {\zeta C_{i} + \left( {1 + \zeta z} \right)D_{i} + 2F_{i} } \right]e^{\zeta z} } \right\}d_{\zeta } ,$$

where, $A_{i}$, $B_{i}$, $C_{i}$, $D_{i}$, $E_{i}$ and $F_{i}$ are constants solved by the following procedures:

When the load acting on the top surface is $p_{H} \left( r \right)$, the boundary conditions for the upper surface $\left( {z = 0} \right)$ are:

11 $$\begin{gathered} \tau_{zr\left( 1 \right)}^{L} = p_{H} \left( r \right)\cos \theta \hfill \\ \sigma_{zr\left( 1 \right)}^{L} = 0, \hfill \\ \end{gathered}$$

where, subscript (1) represents the first layer.

Interfacial bonding conditions for the 3-layered continuous system are: when $\left( {z = h_{1} } \right)$, $\sigma_{z(1)}^{L} = \sigma_{z(2)}^{L} , \, \tau_{zr(2)}^{L} = 0$; when $\left( {z = h_{2} } \right)$, $\sigma_{z(2)}^{L} = \sigma_{z(3)}^{L} , \, \tau_{zr(2)}^{L} = \tau_{zr(3)}^{L}$.

(3) Double circular load.

**Figure 2:**
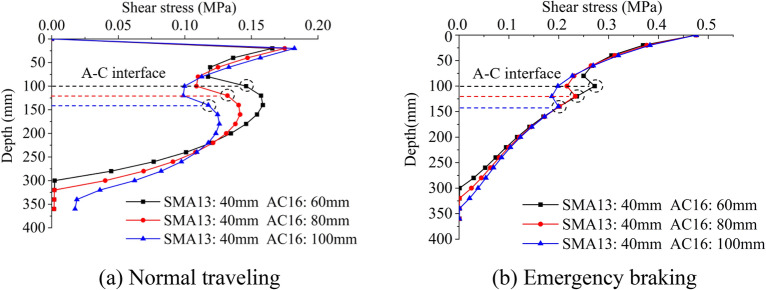
The evolution process of shear stress with depth under different pavement layer thicknesses.

Vehicle wheel load acting on pavement surface is regarded as double circular load (as shown in Fig. 2), and thus the total stresses are expressed as:

12 $$\begin{gathered} \delta_{z} = \delta_{z}^{V} + \delta_{z}^{V} \hfill \\ \tau_{z} = \tau_{zr}^{V} + \tau^{L}_{zr} . \hfill \\ \end{gathered}$$

### Influencing factors

Shear properties on the interfacial layer between the asphalt overlay and the old PCC slab are influenced by several factors. In this section, four factors are investigated, including asphalt overlay thickness, the friction coefficient of wearing course, vehicle load, and bonding situation of the tack coat. The situations of normal driving and emergency braking are considered. The calculation is performed with the multilayer elastic theory.

#### Asphalt overlay thickness

To estimate the effect of asphalt overlay thickness on the mechanical properties of the interfacial layer, the thickness of the asphalt overlay is a variable. Due to the pavement repair practice in the Chongqing region, the thickness of the SMA–13 layer is 40 mm, while the AC–16I layer can be 60 mm, 80 mm, or 100 mm. Other parameters are listed in Table 1. The Elastic Modulus and Poisson's Ratio remained unchanged in the subsequent studies. The friction coefficient of the wearing course is 0.7, the vehicle load is standard load, and the interface is fully-bonded.

**Table 1:** Physical and mechanical parameters of each layer.

Composite	Abbreviation	Thickness h /(mm)	Elastic modulus E /(MPa)	Poisson’s ratio $\mu$
SMA-13	S	40	1400	0.35
AC-16I	A	60/80/100	1200	0.35
C30	C	200	30,000	0.2
Soil base		–	200	0.3

*C30 C30 cement concrete.

The evolution characteristics of shear stress along pavement depth when a vehicle travels and urgently brakes are shown in Fig. 2. When a vehicle travels, the shear stress of each layer dramatically fluctuates. As the depth increases, the shear stress fluctuates, increases first, then decreases, increases and finally damping at 300 mm. The maximum shear stress occurs among the range of SMA material. The local extremums occur inside the PCC materials instead of the A–C interface. However, shear stress on A–C interface is also large enough to be non-negligible, and these values decreases as the thickness of the AC-16I overlay increases. When a vehicle urgently brakes, the evolution trend of shear stress along the thickness is quite different. The maximum shear stress is approximately 0.48 MPa, occurring on the pavement surface. The shear stress decreases as the depth increases, and local extremums occur on the A-C interface. Similarly, the shear stress on the A-C interface slightly increases as the thickness of the AC–16I overlay increases. Asphalt thickness has greater influences on the normal travelling conditions than the emergency braking condition.

Thus, when the thickness of the asphalt overlay is 100 mm, i.e., the SMA-13 layer is 40 mm and the AC–16I overlay is 60 mm, the shear stress on the A–C interface is the highest.

#### Friction coefficient of the wearing course

As we know, there is rolling friction between the vehicle wheel and the ground when traveling, while in the case of emergency braking, it is sliding friction. When the vehicle normally travels, the wearing course has little effect on shear stress as the rolling friction coefficient is much smaller than the sliding friction coefficient. Therefore, only the kinetic sliding friction coefficient *f *of asphalt, usually altering from 0.3 to 0.7, is considered during emergency braking. Here, *f* = 0.3, 0.5, 0.7, respectively. The worst case of 40 mm SMA-13 layer and 60 mm AC-16I overlay is studied. The vehicle load is standard load, and the interface is fully-bonded.

**Figure 3:**
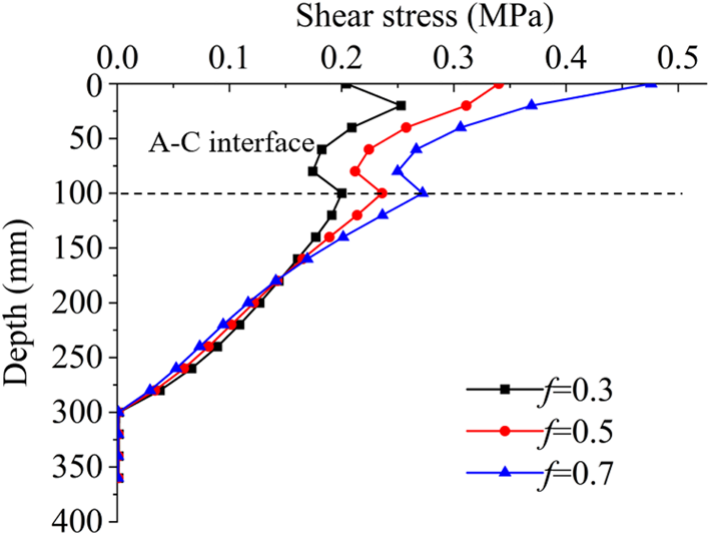
The evolution process of shear stress with depth under different friction coefficients of wearing course surface.

Figure 3 shows the influences of the friction coefficient of the wearing course on shear stress. The shear stress increases as the friction coefficient increases. The friction coefficient of the wearing course decides the longitudinal vehicle load. The rougher the ground surface is, the higher the maximum shear stress on the A–C interfacial layer is.

#### Vehicle load

Since distresses usually occur in the overloading road segment, the influence of vertical vehicle load on the distribution of shear stress is investigated. One axle with four wheels weighs 100 kN, and the vehicle is approximately 15 tons (t). This weight (100 kN) is defined as standard load, corresponding to 0.7 MPa acted on the pavement surface (Specifications for Design of Highway Asphalt Pavement, 2017)^17^ The shear stress distribution of vehicle standard load, overloading 50% (1.05 MPa), and overloading 100% (1.4 MPa) are compared. The thickness of the SMA-13 layer is 40 mm, the AC-16I overlay is 60 mm, the friction coefficient is 0.7, and the interface is fully-bonded.

**Figure 4:**
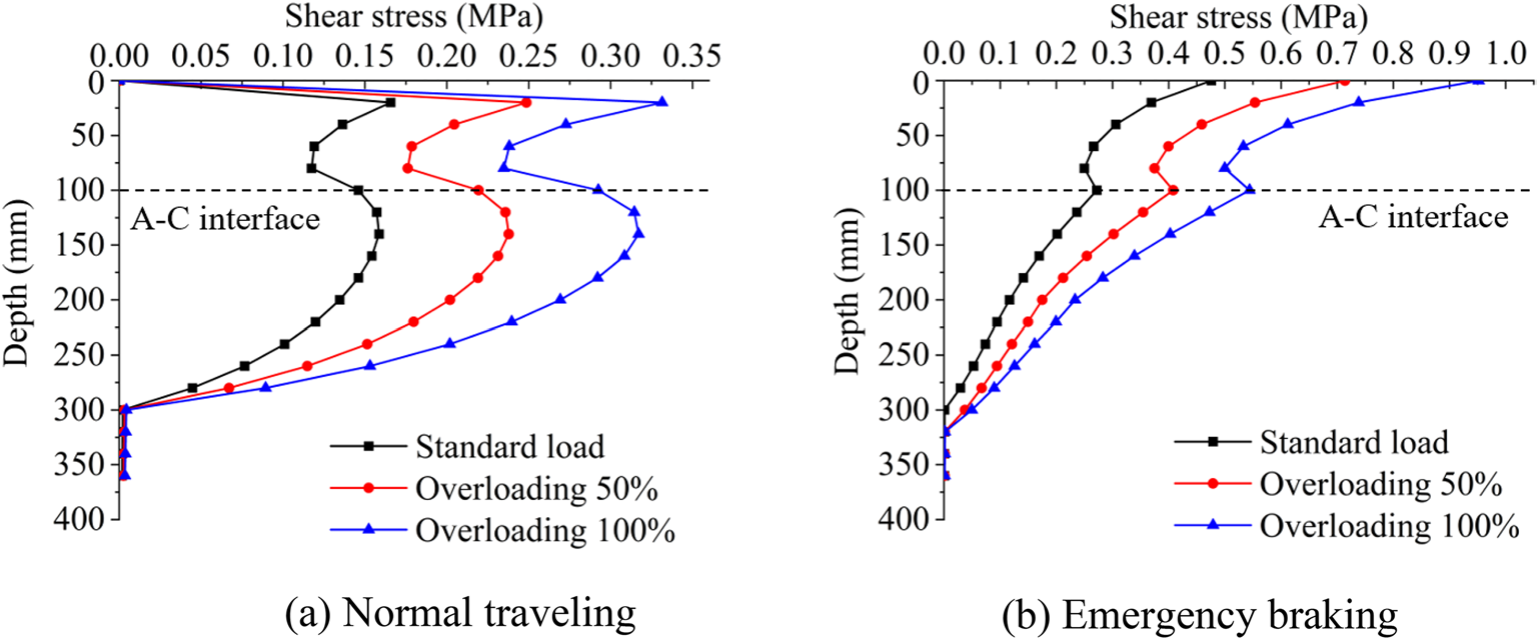
The evolution process of shear stress with depth under different vehicle loads.

Figure 4 shows the influences of vehicle load on shear stress under normal traveling and emergency braking. The shear stress on the A–C interface when a vehicle is emergency braking is always higher than that when a vehicle is normal traveling. The shear stress on the interface is highest when an overloading 100% vehicle is urgently braking.

#### Interfacial bonding condition

There is not only frictional resistance on interfacial layers under vehicle load, but also relative horizontal displacement. Goodman^19^ proposed a model to deal with the discontinuities in rock mass by treating the rock mass as a system of blocks and links, the shear stress on discontinuities surface is linearly related to horizontal displacement. Learn from Goodman’s model, when the relative horizontal displacement between the upper and lower layers is ∆*u*, the shear stress on the interfacial layer of two materials is $\tau = K\Delta u$. The coefficient $K$ is appropriate for describing the bonding state on the interfacial layer. When unit relative displacement takes place between the upper and lower layers of the road surface, the shear stress equals $K$. The larger the coefficient $K$ is, the better the interfacial bonding is, and the interface tends to be fully bonded and vice versa. In most studies, layers are treated fully bonded (*K* tends to infinity) or completely unbounded ($K = 0$) (Diakhate et al. 2006; Alae et al. 2020)^20,21^. Semi-bonded ($0 < K < \infty$) is an intermediate state. The aforementioned three interfacial bonding conditions are considered.

**Figure 5:**
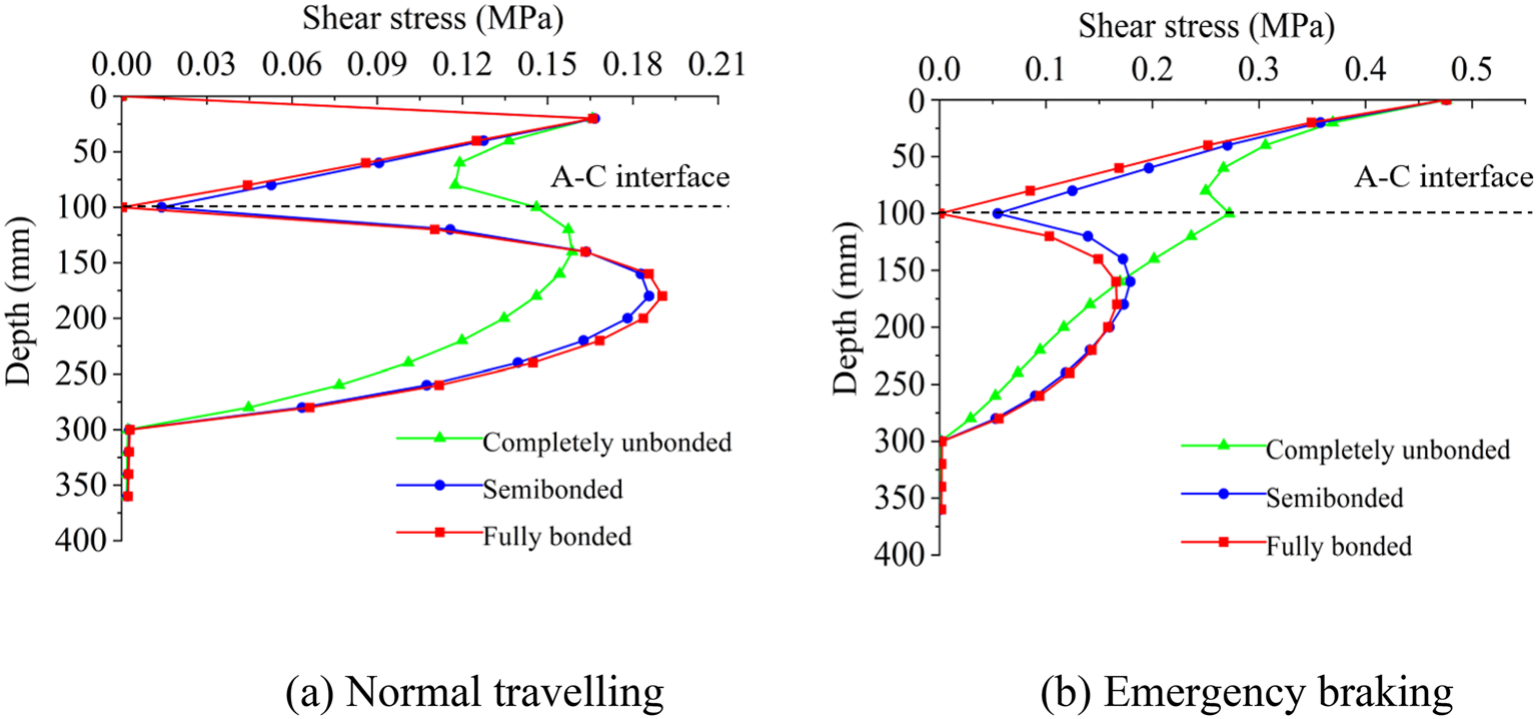
The evolution process of shear stress with depth under different interfacial bonding conditions.

The thickness of the SMA-13 layer is 40 mm, the AC-16I overlay is 60 mm, the friction coefficient is 0.7, and standard load is studied. Figure 5 shows the influences of interfacial bonding on the distribution of shear stress. For the cases of semi-bonded and fully-bonded, the changing trends of shear stress are similar. The values of the shear stress are relatively uniform when the cohesive coefficient on the interlayer is high, and the values of maximum shear stress change sharply when the cohesive coefficient is small. The higher the cohesive coefficient *K* is, the smaller the shear stress on the asphalt overlay and concrete slab is.

In summary, the shear stress on the A–C interface increases with the increasing friction coefficient on the wearing course, vehicle load, and interfacial bonding condition, while it decreases with the increasing asphalt overlay thickness. Vehicle load is an essential factor when fully bonded to find the critical thickness range of the asphalt overlay.

## Experimental methods

Laboratory direct shear tests are performed to obtain the shear strength of the asphalt materials, and the shear strength of the layer between asphalt and cement concrete.

### Material and grouping

As shown in Table 2, five kinds of materials in eight groups are involved. The samples in groups 1 and 4 constitute SMA–13, binder and C30 cement concrete, shorted as S–C. The samples in groups 2 and 5 constitute AC-16I, binder, C30 cement concrete, shorted as A–C. The samples in groups 3 and 6 constitute SMA-13, binder and AC-16I, shorted as S–A. Two kinds of binders, the modified emulsion asphalt (E. A) and epoxy resin adhesive (E. R), are tested. Noted that only one kind of binder is used in one sample.

**Table 2:** Grouping and results in the direct shear tests.

Group	Material	Code	Binder	Interfacial frictional angle $\varphi$ (°)	cohesive force $c$ (${\text{kN}} \cdot {\text{m}}^{ - 2}$)
1	SMA–13, C30	S–C	E.A	27.45	0.12
2	AC–16I, C30	A–C		33.29	0.08
3	SMA–13, AC–16I	S–A		31.55	0.13
4	SMA–13, C30	S–C	E.R	42.22	0.13
5	AC–16I, C30	A–C		44.80	0.28
6	SMA–13, AC–16I	S–A		23.11	0.49
7	SMA	–	–	48.41	0.28
8	AC	–	–	50.89	0.31

### Facility and sample

To simulate the serviceability of asphalt pavements, the concrete is derived from a construction site, SMA–13 and AC–16I are derived from an asphalt material supply station. The particles larger than 4.75 mm occupy 57.7% and 31.9% in SMA–13 and AC–16I, respectively. The asphalt contents in these two kinds of materials are 4.5% and 6.1%, respectively.

**Figure 6:**
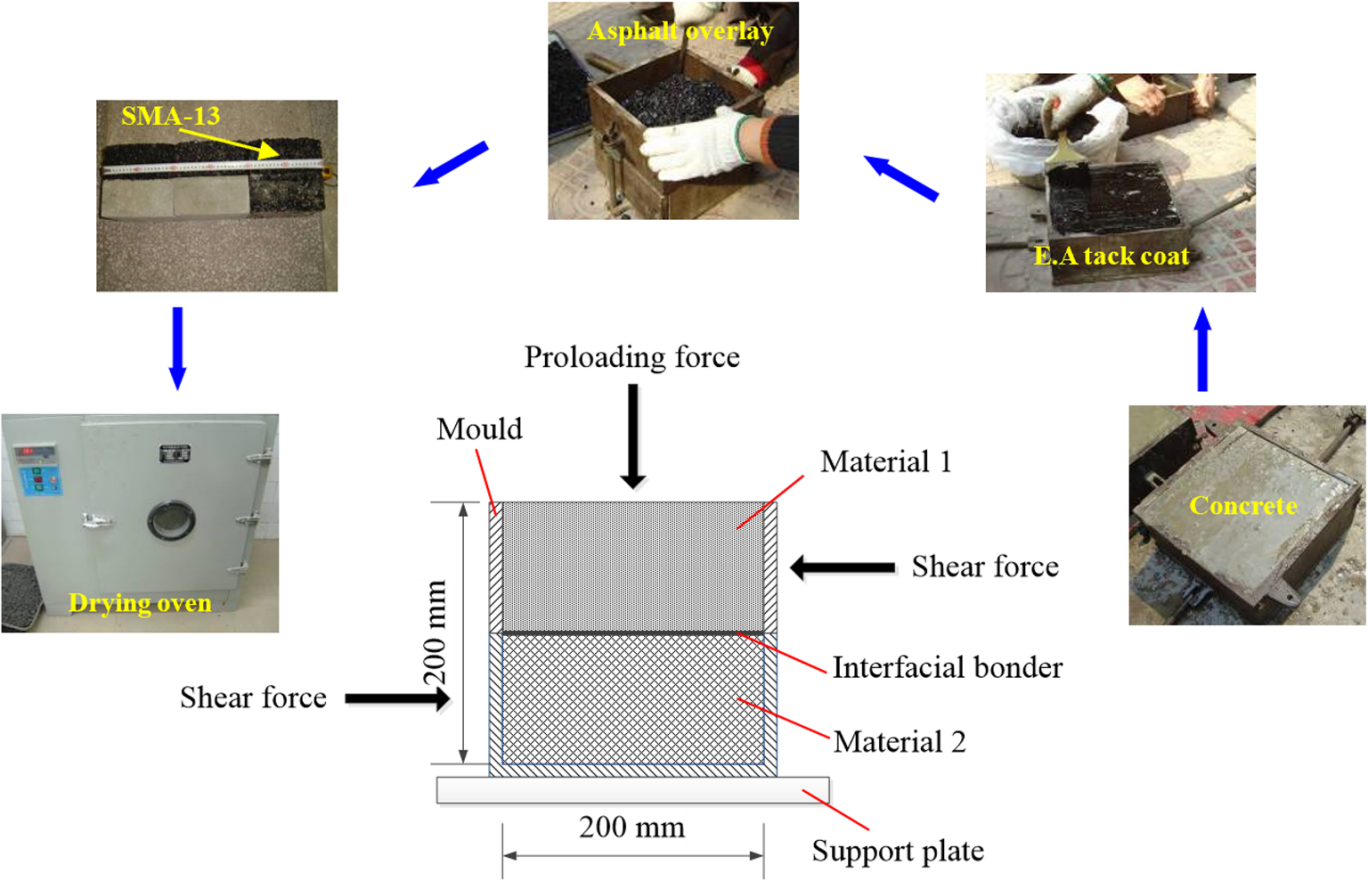
Experimental process and facilities of the sample.

The experiment process and facilities are shown in Fig. 6. The rock direct shear apparatus was modified to fit the 200 mm × 200 mm × 200 mm mould, and to guarantee shear failure takes place on the interface of two materials.

Plenty of research has reported that the moisture and temperature in the curing process greatly influence the shear properties between layers^22,23^. The climate in Chongqing is moist, and the highest temperature can be up to 40 ℃. The moisture and high-temperature effects are taken into account in the direct shear test. The samples were maintained in water for seven days and stored outdoors for three months. Then, they were placed in a drying oven set to 55℃. Direct shear tests were immediately conducted after the sample was taken out from the drying oven.

The normal and shear forces were imposed. The shear force increased at an interval of 10% estimated maximum shear force each time. When shear deformation induced by the latter shear force is greater than 1.5 times that induced by the former shear force, then the imposed shear force is halved until the sample fails.

### Test results

**Figure 7:**
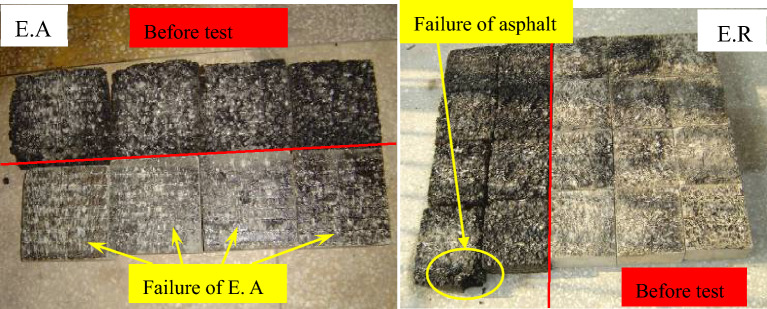
Shear damage phenomenon.

**Figure 8:**
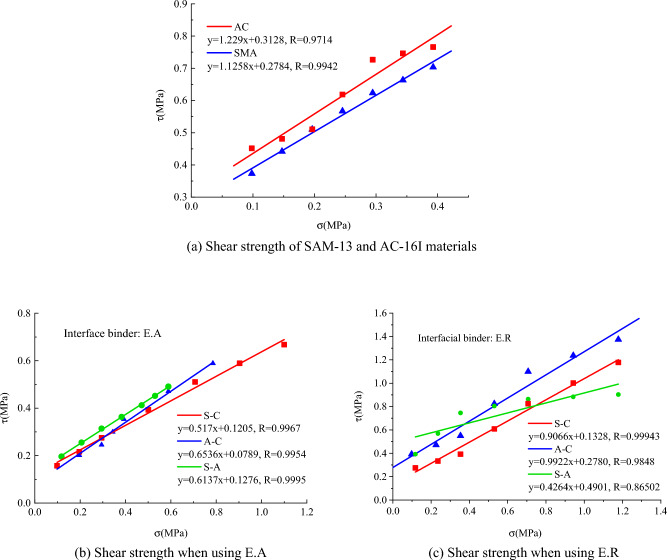
Shear strength testing results of the samples.

Figure 7 shows the failure phenomena of the samples. When using E. A as binder, the sample is shear-damaged on the binder layer, and the E. A is exposed. When using E. R as binder, the asphalt surface course is damaged. The shear failure of asphalt pavement with E. A is caused by the insufficient binding capacity of E. A. However, the situation with E. R is different as E. R is strong enough, causing damage to the asphalt overlay. The shear strength of these samples is summarized in Fig. 8 and listed in Table 2.

The shear strength of asphalt and PCC slab interface meets with the Mohr–Coulomb criterion, so the parameters cohesion *c* and friction angle φ can be used in describing interfacial shear resistance capacity. The shear strength parameters are acquired. It is found that: (1) The shear strength of SMA–13 and AC–16I is higher than that of binder course among SMA–13, AC–16I, and concrete; (2) The shear strength of S–A binder course is higher than that of S–C and A–C. Damage of multi-layered pavement structure tends to take place on the interlayer between asphalt and old PCC slab rather than other places; (3) The shear strength of S–C interface is lower than that of A–C interface. AC–16I is more suitable for bonding cement than SMA–13.

### Shear envelope

Shear strength envelope curves can effectively reflect the shear strength of SMA–13, AC–16I, PCC slab and their interfacial layers. The envelop curves can be determined with the Mohr–Coulomb criterion:

8 $$\tau { = }\sigma \tan \varphi + c,$$

**Figure 9:**
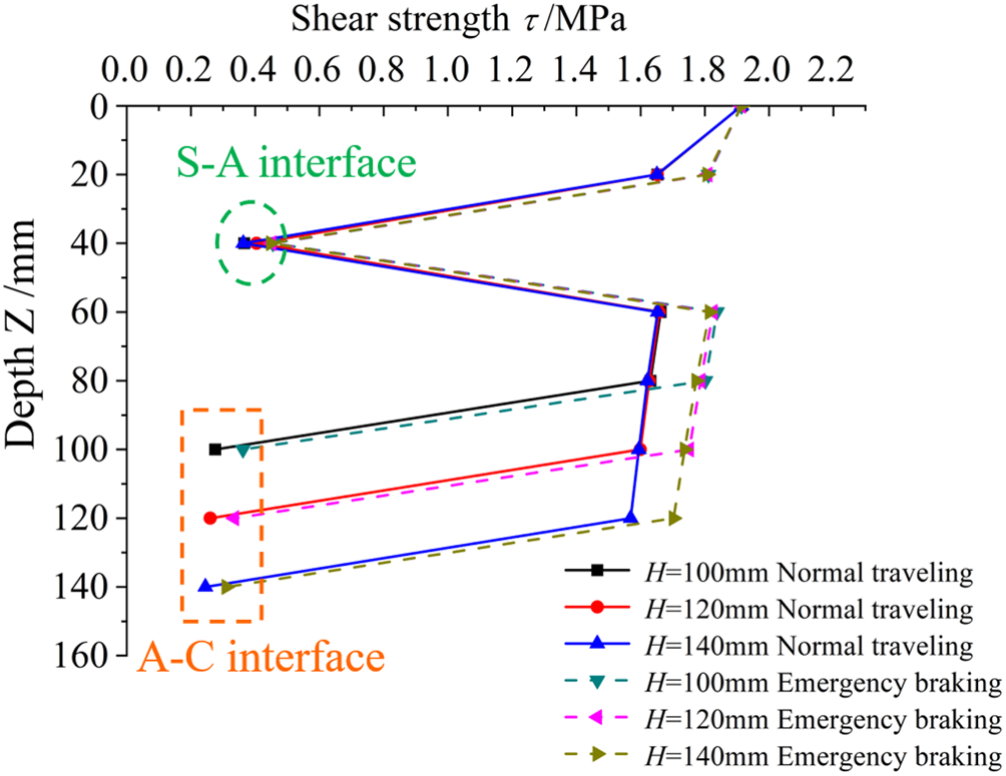
Shear envelope curves.

Taking the disadvantageous E. A as the binder. When the vehicle is normally traveling and emergently brake, relationship between shear strength distribution and asphalt thicknesses is established, as shown in Fig. 9. The shear strength of SMA–13 and AC–16I are much higher than the shear strength on S–A and A–C bonding layers, indicating that it is the shear strength on interface controlling the shear mechanics of the whole asphalt overlay system. What’s more, it can be seen that both asphalt thickness and traveling conditions have less influence on the distribution patterns and values of shear strength, while they significantly impact the shear stress. This implies establishing a thickness calculation method for asphalt overlay by comparing the shear strength and shear stress, which is analyzed and discussed in “Analysis and discussion”.

## Analysis and discussion

In this section, a design method for asphalt overlay thickness is proposed based on the shear performance of the interlayer between the asphalt overlay and the old PCC slab. The designed asphalt thickness is advisable only when the shear stress on the interface on asphalt and PCC slab is smaller than the shear strength. The shear stress is calculated according to the method in “Mechanics theory”, and the shear strength is obtained from the shear envelope in “Experimental methods”.

**Figure 10:**
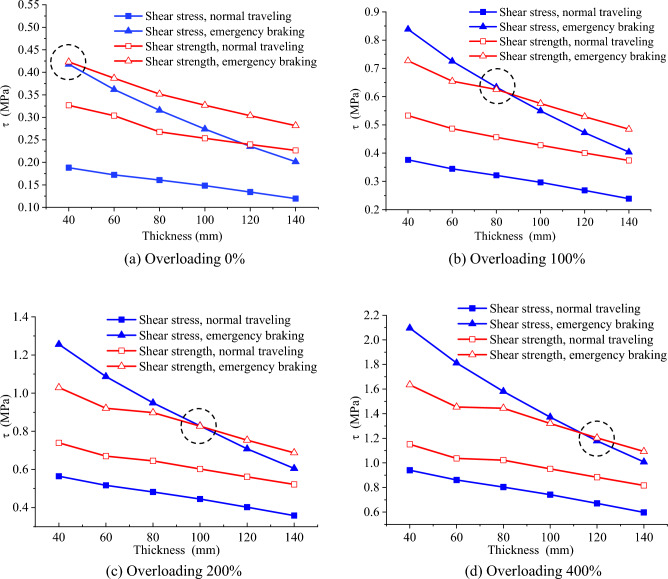
Comparison of shear strength and shear stress under different vertical loads. The blue lines represent the shear stress, and the red lines represent shear strength. The black dashed circle indicate the point where the shear stress exceeds the shear strength.

Figure 10 gives an example of designing the asphalt overlay thickness. The vehicle load reaches up to overloading 400% at an interval of 100%. The conditions include normal traveling and emergency braking, and the 40–80 mm single-layered asphalt overlay and 80–140 mm double-layered/multi-layered pavement structures. It is assumed that the requirement of pavement deflection and the bending tensile stress are satisfied. The interlayers are fully-bonded, and the friction coefficient is 0.7 when emergency braking.

When the vehicle normally travels and the vehicle load reaches up to overloading 400%, the thickness range of 40–140 mm is capable of resisting the shear stress on the interlayers. The maximum shear stress is smaller than the shear strength, and horizontal shear damage will not occur. The 40–140 mm thickness asphalt overlay for the smooth traffic section can bear any vehicle.

The specific thickness ranges are listed in Table 3. In Fig. 10a, when an overloading 0% vehicle emergency braking, the maximum shear stress and shear strength are very close at the thickness of 40 mm, and horizontal shear damage may occur. When the thickness is 50–140 mm, the maximum shear stress is smaller than the shear envelope, and the interlayers are relatively safe from shear damage. This indicates that 40–50 mm thickness is capable of small and medium vehicles at any section of the road. Note that parking stations and corners are excluded, and similarly in the following analysis. The pavement thickness of 60–90 mm can meet the need for large vehicles. According to the traffic situation in Chongqing, it is defined that the weight of a small car is lighter than 5 t, the weight of a medium car is higher than 5 t and smaller than 15 t, and a large car is heavier than 15 t.

**Table 3:** Thickness range of the asphalt overlay /mm.

	Small vehicles (weight < 5 t)	Medium vehicles (5 t < weight < 15 t)	Large vehicles (weight > 15 t)
Overloading 0%	40–50		60–90
Overloading 100%	80–90		100–140
Overloading 200%	100–140		
Overloading 400%	120–140		

In Fig. 10b, when a 100% overloading vehicle suddenly brakes, if the pavement thickness is no more than 80 mm, the maximum shear stress is higher than the shear envelope, and horizontal shear failure will occur. The pavement can avoid shear failure when the thickness range is 90–140 mm. This indicates that the pavement with 80 mm thickness is capable of small/medium vehicles in any section of the road. The asphalt overlay with 100–140 mm thickness is capable of large vehicles in any section.

In Fig. 10c, when a 200% overloaded vehicle suddenly brakes and the pavement thickness is less than 100 mm, the maximum shear stress exceeds the shear envelope, and thus the horizontal shear damage may occur. This indicates that the pavement with 100–140 mm thickness is capable of a large vehicle in any section of the road.

In Fig. 10d, when a 400% overloaded vehicle suddenly brakes and the pavement thickness is less than about 110 mm, the maximum shear stress exceeds the shear envelope, and thus the horizontal shear damage may occur. This indicates that the pavement with 120–140 mm thickness is capable of any vehicle in any section of the road.

According to the results, the pavement thickness $H$ can be designed in the cases of a single-layer system or a double-layer system. The single-layer system of approximately 50 mm is suitable for the road sections with small traffic and small/medium vehicles. The double-layer system with 80–100 mm thickness is suitable for heavy roads with large traffic and large/medium vehicles. The double-layer system with 100–120 mm thickness is suitable for large vehicles. If severe super-overloaded vehicles are in some road sections, a three-layered pavement structure of about 140 mm is suggested. The results are consistent with Wang^16^, who suggested that the optimal thickness range of asphalt layer should be set to 100–200 mm based on energy dissipation principle. Proposing thickness range by considering different traffic conditions is more reasonable, and thus to achieve the goal of energy conservation and emission reduction. For areas with sizeable local shear stress, such as bus stations, steep slopes and sharp turns, a thicker asphalt layer can be used to decrease the shear stress or E. R is suggested to increase the shear strength.

## Conclusions

A simple calculation method for the thickness of the asphalt overlay on the old PCC slab is proposed by comparing the shear stress distribution and shear strength, in which the shear stress is obtained by multilayer elastic theory, and the shear strength of the asphalt and concert interlayer is established by shear envelope. The study support following conclusions:

- (1) Shear stress distribution patterns are quite different between the normal travel and emergency braking. In both conditions, the shear stress on the asphalt and PCC slab interface increased with the increasing friction coefficient on wearing course, vehicle load, and interfacial bonding condition, while it decreased with the increasing asphalt overlay thickness.
- (2) The shear strength values of materials and interlayers meet the Mohr–Coulomb criterion, and the shear strength envelop curves were established via direct shear tests of double-layered pavement model. The shear strength on asphalt and concert interface controls the shear mechanics of the whole asphalt overlay system.
- (3) The asphalt thickness design should consider the traffic load and driving conditions (normal travel or emergency braking), the thickness range from 50 to 140 mm is suggested according to different travel conditions. For areas with sizeable local shear stress, such as bus stations, steep slopes and sharp turns, a thicker asphalt layer or E. R is recommended in restoration. If the minimum recommended thickness is not allowed in some situations, such as a load-limited bridge, it was suggested that E. R should be used as a binder due to its stronger adhesive properties.

## Data Availability

The datasets used and/or analysed during the current study available from the corresponding author on reasonable request.
